# Establishment of a Lung Metastatic Breast Tumor Xenograft Model in Nude Rats

**DOI:** 10.1371/journal.pone.0097950

**Published:** 2014-05-16

**Authors:** Joris Tchouala Nofiele, Hai-Ling Margaret Cheng

**Affiliations:** 1 The Research Institute and Diagnostic Imaging, Hospital for Sick Children, Toronto, Ontario, Canada; 2 Department of Medical Biophysics, University of Toronto, Toronto, Ontario, Canada; 3 Leslie Dan Faculty of Pharmacy, University of Toronto, Toronto, Ontario, Canada; 4 The Institute of Biomaterials & Biomedical Engineering, University of Toronto, Toronto, Ontario, Canada; University of Texas Health Science Center, United States of America

## Abstract

**Objective:**

Larger animal models provide relevant tumor burden in the development of advanced clinical imaging methods for non-invasive cancer detection and diagnosis, and are especially valuable for studying metastatic disease. Most available experimental models, however, are based on immune-compromised mice. To lay the foundation for studying spontaneous metastasis using non-invasive magnetic resonance imaging (MRI), this study aims to establish a highly metastatic breast cancer xenograft model in nude rats.

**Materials and Methods:**

A highly metastatic variant of human adenocarcinoma MDA-MB-231 known as LM2 was inoculated into nude rats. Orthotopic and subcutaneous (flank) sites were compared, with half of the orthotopic injections guided by ultrasound imaging. MRI with gadolinium contrast administration was performed weekly beginning on Day 6 and ending on Day 104. Excised tumors were assessed on histology using hematoxylin and eosin and CD34. Fisher's exact test was used to compare successful tumor induction amongst different inoculation methods.

**Results:**

Primary LM2 tumors were established orthotopically in all cases under ultrasound-guided injection, and none otherwise (*p* = 0.0028). Contrast-enhanced MRI revealed rapidly progressing tumors that reached critical size (15 mm diameter) in 2 to 3 weeks after inoculation. MRI and histology findings were consistent: LM2 tumors were characterized by low vascularity confined to the tumor rim and large necrotic cores with increasing interstitial fluid pressure.

**Conclusions:**

The metastatic LM2 breast tumor model was successfully established in the mammary fat pads of nude rats, using ultrasound needle guidance as a non-invasive alternative to surgery. This platform lays the foundation for future development and application of MRI to study spontaneous metastasis and different stages throughout the metastatic cascade.

## Introduction

Current medical imaging technologies play a critical role in the diagnosis and management of cancer patients. Magnetic resonance imaging (MRI), for example, is a non-irradiative whole-body imaging platform that enables accurate localization and sensitive delineation of tumor masses, characterization of tumor vascularity, and non-invasive monitoring of treatment effects [1]–[3]. Imaging also plays a key role in the clinical translation of new treatment paradigms, as a non-invasive method is ultimately desired for monitoring the progress of patients receiving therapy. However, imaging technologies that are used to test the efficacy and safety of new investigational drugs in preclinical mouse models are not easily translated to human imaging. In other words, there is a gap in the technologies developed for preclinical testing and those ultimately required in the clinic. One way to close this gap is to use larger animal models. Not only can larger animals be imaged at spatial resolutions easily translated to human imaging, but also they allow a closer approximation to tumor masses and metastatic sites found in patients. Increasing recent efforts toward developing tumor models, such as hepatocellular carcinomas [4]–[6], in rats nods to the advantages of using larger animals.

In the study of breast cancer, as in other cancers, developing models for metastatic disease is very important, as it remains the principal cause of mortality. A highly metastatic variant of the commonly used hormone-independent MDA-MB-231 human breast adenocarcinoma was developed by Munoz et al. using serial selection for metastasis in the lungs [7]. This mouse xenograft model, known as 231/LM2-4, was shown to establish macroscopic lung nodules only two months after resection of the primary tumor [7]. In the handful of studies on this tumor model to date, mice have been used exclusively to understand breast tumorigenesis and metastasis [8] and to study the efficacy of anti-vascular agents [9]. Establishment of this pro-metastatic variant in a larger animal model has not been reported.

In this study, our aim was to establish the highly metastatic breast cancer xenograft model 231/LM2-4 in nude rats. Using xenografts to mimic breast cancer in rats is not common, and investigations in rats over the past 50 years have been restricted mainly to chemical carcinogenesis [10]. However, commonly used chemical agents such as 7,12-dimethylbenz(a)anthracene (DMBA) or N-nitroso-N-methylurea (NMU) induce mainly hormone-dependent adenocarcinomas [11]. Having the ability to also investigate hormone-independent tumors in rats would be beneficial, in light of the histological similarities that have been demonstrated between rat mammary tumors and human breast cancers [11], [12]. In this pilot study, we investigate two different methods for generating xenografts and perform MRI to non-invasively monitor and characterize tumor development in vivo.

## Materials and Methods

### Nude Rats

All procedures were approved by the Hospital for Sick Children Animal Care Committee (protocol #22918) and conducted in accordance with the “Animals for Research Act of Ontario” and “Guidelines of the Canadian Council on Animal Care”. Thirteen healthy 6-weeks-old female immunodeficient rats (Harlan Laboratories) were used in this study, and all efforts were made to minimize distress.

### Tumor Cell Line and Culture

The 231/LM2-4 breast cancer cell line, hereafter referred to as LM2, is a highly metastatic variant of the human adenocarcinoma MDA-MB-231, a triple negative breast cancer (i.e. negative for estrogen receptor, progesterone receptor, and HER2/neu), and was obtained after two rounds of metastasis selection in mice [7]. Cells were grown at 37°C with 5% CO_2_ in 1640-RPMI medium (Sigma-Aldrich Canada Inc., Oakville, ON, Canada) supplemented with 10% fetal bovine serum and 0.5% penicillin streptomycin. Fresh supplemented medium was added every other day. Cells were harvested by washing 80% confluent flasks with phosphate buffered saline (PBS) and adding 0.05% trypsin EDTA (Gibco, Carlsbad, CA, USA) to detach cells. Cells were then harvested, counted, and resuspended in PBS at a concentration of 4×10^7^ cells/mL.

### Tumor Cell Inoculation

**Figure 1:**
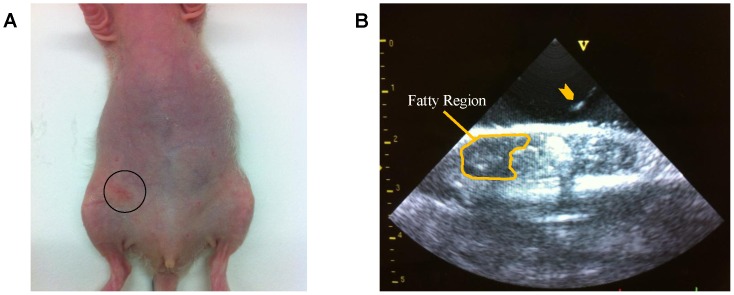
Cell inoculation under ultrasound image guidance. (A) Cancer cells were injected through the skin (circle) and into the lower mammary fat pad under ultrasound image guidance. (B) Ultrasound image showing needle tip (arrowhead) and mammary fat pad.

Three different methods for establishing xenografts were tested. In one group of rats (*n* = 5), cells were prepared in solution containing 25% matrigel (v/v) and approximately 1×10^7^ cells were injected subcutaneously into the flank. In a second group of rats (*n* = 4), approximately 1×10^7^ cells were injected into the mammary fat pad while the animal was anesthetized with 1.5% isoflurane. A 25G needle was used to minimize trauma to the cells. Injection into the mammary fat pad was performed under real-time ultrasound image guidance, using a GE 10.0FPA 10 MHz pediatric cardiac transducer (GE Healthcare) and a GE VingMed Ultrasound system. Guidance into the fat pad was possible due to strong signal reflection from the needle. Figure 1 shows where the needle was inserted in the rat and how ultrasound image guidance was performed. The needle was placed in the plane of the ultrasound beam and directed at an angle to ensure accurate penetration into the fat pad. This type of image guidance essentially allows one to accurately transplant cells in the desired target without resorting to invasive surgery, which is the standard for orthotopic inoculation. A third group of rats (*n* = 4) was also injected orthotopically but without ultrasound image guidance or surgery, thus requiring a certain degree of approximation of the needle penetration depth.

### Magnetic Resonance Imaging

Beginning on Day 6 after cancer cell inoculation, rats were imaged weekly a 3-Tesla clinical MR scanner, using an eight-channel wrist coil for signal detection. Rats were induced on 2% isoflurane in pure oxygen (2 L/min flow rate) and maintained on 1.5% isoflurane during imaging. Rats were placed prone within the coil, resting on top of a water-blanket maintained at 36 degrees Celsius (HTP-1500, Adriot Medical Systems, Loudon, TN, USA). A 24-gauge angiocath was inserted into the lateral tail vein for contrast injection, and this was connected through a 1-mL line tubing to a 3-way stop-cock through which gadolinium (Gd) and saline could be delivered separately. Gd-DTPA (Magnevist, Bayer) was injected as a bolus at a dose of 0.05 mmol/kg, followed by 2 mL of saline.

In addition to localizer scans, a baseline high-resolution anatomical *T*_1_-weighted spin-echo scan and *T*_1_ map were acquired. The *T*_1_-weighted spin-echo scan used a 2D acquisition with the following parameters: repetition time (TR)  = 724 ms, echo time (TE)  = 14 ms, number of signal averages (NSA)  = 3, 100 mm field-of-view (FOV), twenty 1-mm thick slices, and 0.6×0.6 mm in-plane resolution. *T*_1_ mapping consisted of a 3D spoiled fast field echo (T1-FFE) sequence repeated at flip angles of 2°, 10°, and 20°; other parameters were TR = 6.2 ms, TE = 3.2 ms, NSA = 8, 100 mm FOV, twenty 1-mm thick slices, and 0.6×0.6 mm in-plane resolution [13]. A dynamic contrast-enhanced (DCE) MRI sequence was then run to capture the kinetics of contrast uptake and washout. This employed a keyhole acquisition on a 3D T1-FFE to achieve a temporal resolution of 9.1 s while maintaining a spatial resolution of 0.6×0.6×1 mm over twenty slices. This dynamic sequence was run for 8 min and 52 s, and contrast was injected intravenously as a bolus 2 minutes after the start of the dynamic sequence. Immediately afterwards, the *T*_1_-weighted spin-echo scan and *T*_1_ mapping were repeated.

### Data Analysis

MRI data was transferred to an independent workstation for quantitative data analysis using in-house software developed in Matlab (v.8.1) (MathWorks, Natick, MA).

In-vivo data was first analysed for *T*_1_ relaxation times before contrast injection. *T*_1_ maps were generated by calculating *T*_1_ relaxation times on a pixel-wise basis on every slice in the 3D imaging volume using a previously described method [13]. This allows the conversion of signal intensity at every pixel location to absolute contrast concentration. From the DCE-MRI acquisition, contrast uptake kinetics was then analysed. Based on the calculated baseline *T*_1_ value, the signal intensity time-course at each pixel location was converted to a contrast concentration time-course [14]. The area-under-the-concentration curve was then computed over the first 90 seconds (AUC_90_) after contrast arrival in tissue [15]. The AUC_90_ metric provides semi-quantitative assessment of the vascularity and leakiness of tumor blood vessels and is a mainstay in current clinical practice of DCE-MRI [16]. To appreciate differences in the contrast uptake profile in different tumor regions, regions-of-interest (ROIs) were drawn to encompass the enhancing tumor rim and low-enhancing core; the ROI-averaged signal intensity SI(t) was determined as a function of time over the duration of the DCE-MRI acquisition and then expressed as a relative signal enhancement according to the formula, SI_rel_(t)  =  (SI(t) – SI_base_)/SI_base_×100%, where SI_base_ is the average pre-injection signal.

### Statistical Analysis

Fisher's exact test (two-tailed) was used to determine if the three methods of cell inoculation were different in their ability to successfully establish tumors. This test is useful when sample sizes are small.

### Histology

Tumors were excised anywhere between 2 to 3 weeks post-tumor induction, depending on their growth rate in vivo. Samples were fixed in 10% neutral buffered formalin at room temperature and processed for histopathology. This involved embedding in paraffin, sectioning into 5 µm thick slices, and staining with hematoxylin and eosin (H&E) and CD34. Light microscope images (Olympus BX60) were taken to assess tumor composition, structure, and vascularity.

## Results

### Successful Establishment of Orthotopic LM2 Tumors in Rats

**Figure 2:**
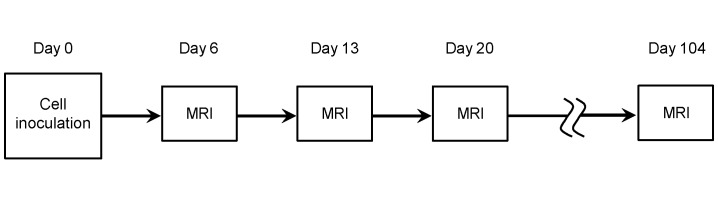
Experimental design. Timeline for cell inoculation and magnetic resonance imaging (MRI).

Tumor induction rate was 100% (4/4) in the orthotopic model when ultrasound image guidance was used and 0% (0/4) when it was not (Table 1). All successfully established tumors demonstrated rapid growth and reached a size of 1.91±0.64 cm^3^ in 2 to 3 weeks after cell inoculation. This result demonstrates the importance of accurately injecting cells within the mammary fat pad and the effectiveness of ultrasound guidance as a non-invasive alternative to surgery for implanting cells in the fat pad. In contrast, none of the rats injected subcutaneously in the flank (0/5) developed tumors; animals in this group were monitored out to 104 days after cell inoculation (Figure 2), and no evidence of tumor development was visible on imaging or on gross dissection. The three methods of inoculation were different in their ability to successfully establish tumors in vivo (*p* = 0.0028).

**Table 1:** Success of establishing LM2 tumor xenografts in nude rats.

Inoculation method	Take rate	Number of animals
Orthotopic under US* needle guidance	100%	4
Orthotopic without US* needle guidance	0%	4
Subcutaneous (flank)	0%	5

*US: ultrasound image.

### Magnetic Resonance Imaging

**Figure 3:**
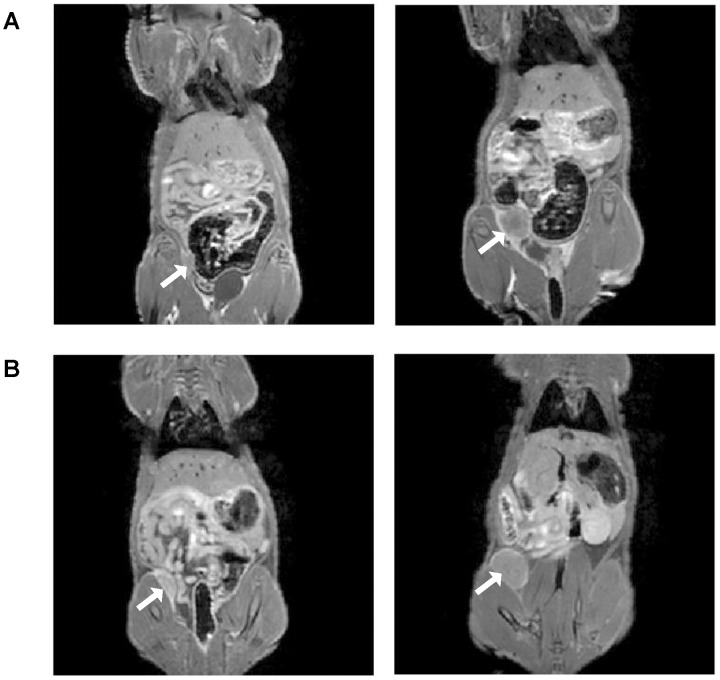
In-vivo gadolinium contrast-enhanced magnetic resonance imaging (MRI) of orthotopic breast tumors in nude rats at 3 Tesla. *T*_1_-weighted spin-echo images acquired post-contrast injection at early and late stages of tumor development are shown in two rats. (A) MRI was acquired 6 days (left) and 20 days (right) after cell inoculation in the mammary fat pad. (B) MRI was acquired 6 days (left) and 13 days (right) after cell inoculation in the mammary fat pad. Arrow points to tumor.

Figure 3 shows in-vivo Gd-enhanced *T*_1_-weighted spin-echo images in two rats bearing orthotopic LM2 tumors. Rapid induction was consistently observed, with tumors averaging 0.30±0.15 cm^3^ in size only 6 days after cell inoculation. Growth rate varied, with most tumors reaching critical size (15 mm diameter) by 20 days (Figure 3A) and one tumor reaching critical size after only 13 days (Figure 3B). In all cases, however, enhancement of the tumor upon Gd administration was modest and was restricted mainly to a thin border surrounding the tumor mass.

**Figure 4:**
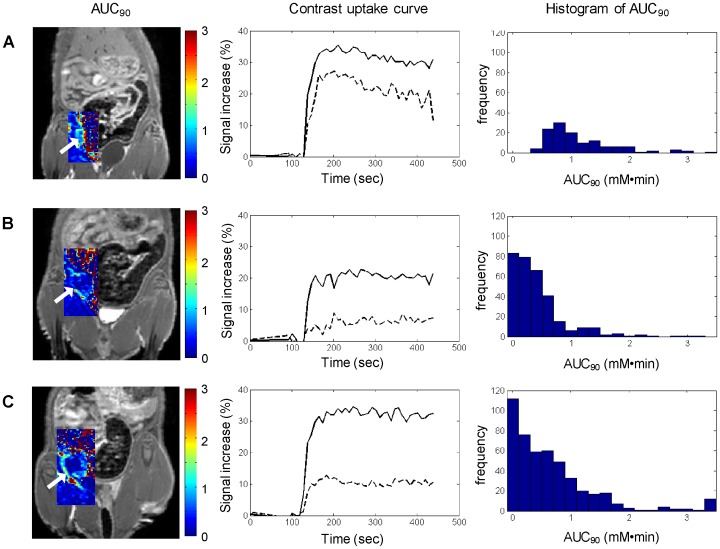
Dynamic contrast-enhanced MRI during tumor progression. (Left column) AUC_90_ maps (mM⋅min) are overlaid on *T*_1_-weighted spin-echo images acquired post-contrast injection (A) 6 days, (B) 13 days, and (C) 20 days after cell inoculation in the mammary fat pad. Arrow points to tumor. (Middle column) Corresponding signal enhancement time-course is shown for the tumor rim (solid line) and tumor core (dashed line). (Right column) Corresponding histograms of AUC_90_ distributions show an evolution toward lower AUC_90_ values.

To quantify DCE-MRI enhancement, Figure 4 superimposes AUC_90_ maps on anatomical *T*_1_-weighted spin-echo images and evaluates changes in contrast kinetics as an orthotopic LM2 tumor develops in vivo. Regardless of the stage of tumor progression, the tumor rim always exhibits higher AUC_90_ values than the tumor core (Figure 4, left column). An important observation, however, is that contrast uptake in the tumor core decreases substantially as the tumor grows in size. This is easily appreciated on signal enhancement profiles over time (Figure 4, middle column). It also underlies a shift in the histogram distribution towards very low AUC_90_ values (Figure 4, right column).

### Histology

**Figure 5:**
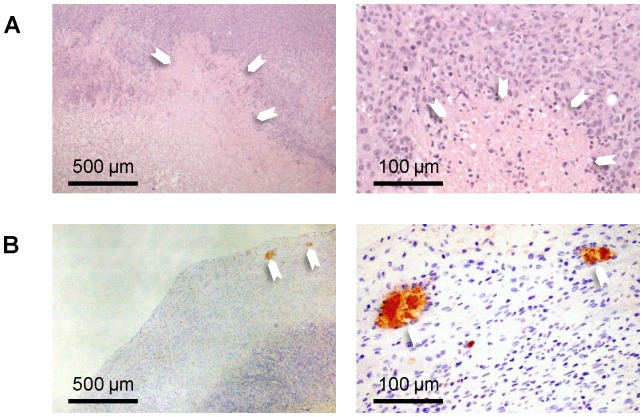
Histology. (A) H&E sections reveal poorly differentiated tumor cells and necrotic areas that are partially or completely devoid of cells (arrowheads). (B) CD34 sections show very low vascularity, with blood vessels (arrowheads) confined to the tumor rim. Magnification is ×4 (left column) and ×20 (right column).

Figure 5 shows histological assessment on H&E and CD34. Morphologically, the LM2 tumor presented as a solid mass comprised of poorly differentiated cancer cells. Very low vascularity was observed and blood vessels that were present were confined mainly to the tumor periphery. Central necrosis was observed in every case and was surrounded by a viable tumor region. In some tumors, completely acellular necrotic areas were also identified.

## Discussion

The use of larger animal models such as the nude rat provides tumor burden that more closely resembles human cancer and is therefore invaluable in the development of advanced non-invasive imaging technologies for clinical application. Image spatial resolutions adequate for sensitive detection and detailed delineation in a rat can be translated to imaging metastasis in humans, potentially enabling very early cancer detection of both primary and metastatic lesions. However, although immunodeficient strains have been developed from rats, they are not widely used in cancer studies. In this study, we investigated whether or not the LM2 xenograft model, which is a highly metastatic variant of human adenocarcinoma MDA-MB-231, could be successfully established in nude rats. It was shown that the site of implantation dictated the take rate, with 100% success achieved orthotopically when cell injection into the mammary fat pad was guided by ultrasound and zero success subcutaneously in the flank. MRI monitoring of tumor development in vivo demonstrated very rapid progression, with tumors reaching critical size in only 2 to 3 weeks after inoculation. DCE-MRI of Gd contrast kinetics revealed a hypovascular core that increased in size and decreased in contrast uptake as the tumor grew. Histological assessment on H&E and CD34 revealed low vascularity confined mainly to the tumor rim, which is in agreement with DCE-MRI findings. Central necrosis was also observed, which is consistent with hypoxia as a result of rapid growth and exacerbated by the low vascularity of this tumor model.

The usefulness of the LM2 tumor model is rooted in its highly metastatic nature and the potential to generate spontaneous metastasis in the rat as it does in mice [7]. In this study, we have achieved the important first step of establishing a method to successfully xenograft a primary orthotopic LM2 tumor, which opens the opportunity to study the metastatic process and many aspects of the metastatic cascade that are bypassed using experimental metastasis models (i.e. injecting tumor cells directly to the systemic circulation). The most important advantage of a spontaneous metastasis model is its ability to capture all the early steps in the cascade that are eliminated through experimental metastasis models, including the critical step of how tumor cells break free from the primary site and associate with host cells before residing at distant locations. Due to the rapid growth of the LM2 model, however, metastasis studies will require complete resection of the primary tumor to control morbidity.

A success rate of 100% for establishing an orthotopic tumor is very high, considering that breast cancer is one of the more difficult tumors to establish in experimental animals, including nude mice and SCID mice, with a success of only 7–20% [17] and considering that take rates are even lower in rats. Further testing in a larger number of rats will establish a more accurate take rate, but we expect this figure would remain high given the results attained in this study. As to the inability of the other two methods to establish tumors, there are plausible explanations. The first method, subcutaneous injections in the flank, is a common approach in mice, and the majority of breast xenograft models in mice are, in fact, subcutaneous. Failure to establish tumors in the rat flank is, therefore, surprising. However, it should be noted that tumor initiation in the flank may not be as rapid as in the mammary fat pad. Where tumor initiation is not rapid, potential differences in immunodeficiency between rats and mice, where it is well known rats are “more leaky”, may result in early elimination of cancer cells by the rat's own immune response before the tumor has an opportunity to grow. The second method, orthotopic injection, is preferred as the mammary fat pad provides a more favorable microenvironment to rapidly initiate tumor growth. However, with this method it is critical that all the cells are deposited within the fat pad – hence, the need for needle guidance, whether by conventional surgery or non-invasively by ultrasound imaging. When injection is performed without guidance, there is the unwanted possibility that some cells will be deposited outside of the fat pad. Between the two alternatives for needle guidance – surgery versus ultrasound imaging – both require familiarity with the respective techniques, but ultrasound offers the advantage of being completely non-invasive.

MRI and histological findings are consistent. Together, they demonstrate a rapidly progressing tumor characterized by very low vascularity confined to the rim and an expanding central necrotic region in which Gd uptake, as measured by AUC_90_, decreases as the tumor grows. There are multiple influences on the uptake of Gd, the primary ones being vascularity and blood vessel leakiness. Therefore, areas with high AUC_90_ correspond to areas where blood vessels are present to deliver Gd to the tumor. However, Gd is an extravascular contrast agent, meaning that it will extravasate from blood vessels and flow into the interstitial space. The transport of Gd through the interstitium is dictated by its own concentration gradient and also by interstitial fluid pressure. If high interstitial fluid pressure is present in the tumor core, the transport of Gd will be slow and will result in low AUC_90_ values. Gross dissection revealed a fluid-filled core in some tumors, which is consistent with high interstitial fluid pressure. This area corresponds both to the low AUC_90_ region on MRI and to the necrotic region on histology. Overall, the MRI and histological findings demonstrate that the LM2 is an orthotopic breast tumor model that requires little vascular support and yet can maintain rapid growth.

Future studies will investigate spontaneous metastasis arising from a primary LM2 breast tumor. The primary lesion will be removed 2 to 3 weeks after inoculation, depending on the growth rate in vivo, and the animal recovered. It is expected that successful xenografts will be established most of the time, given the high take rate achieved in this study. The time to appearance of metastatic lesions will need to be determined and may likely differ from the two months interval reported in mice [7]. These studies will provide a platform for developing and applying new MRI methods for investigating and detecting breast cancer metastasis.

## Conclusions

This study established for the first time in nude rats a highly metastatic orthotopic breast cancer known as LM2. It was shown that success was highly dependent on the site of inoculation, with the orthotopic site yielding 100% take rate while the subcutaneous (flank) site failed to produce xenografts. Monitoring by contrast-enhanced MRI revealed rapid growth in vivo and low uptake of exogenously administered Gd. Assessment on histology demonstrated findings consistent with imaging, including low vascularity confined to the tumor rim and a rapidly developing necrotic core. Establishment of this tumor model lays the foundation for studying spontaneous metastasis models of breast cancer in a larger rat model.
